# Aberrantly Methylated-Differentially Expressed Genes Identify Novel Atherosclerosis Risk Subtypes

**DOI:** 10.3389/fgene.2020.569572

**Published:** 2020-12-14

**Authors:** Yuzhou Xue, Yongzheng Guo, Suxin Luo, Wei Zhou, Jing Xiang, Yuansong Zhu, Zhenxian Xiang, Jian Shen

**Affiliations:** Department of Cardiology, The First Affiliated Hospital of Chongqing Medical University, Chongqing, China

**Keywords:** methylation, differentially expressed gene, lncRNA, atherosclerosis, K-means clustering

## Abstract

Increasing evidence has indicated that modulation of epigenetic mechanisms, especially methylation and long-non-coding RNA (lncRNA) regulation, plays a pivotal role in the process of atherosclerosis; however, few studies focused on revealing the epigenetic-related subgroups during atherosclerotic progression using unsupervised clustering analysis. Hence, we aimed to identify the epigenetics-related differentially expressed genes associated with atherosclerosis subtypes and characterize their clinical utility in atherosclerosis. Eighty samples with expression data (GSE40231) and 49 samples with methylation data (GSE46394) from a large artery plaque were downloaded from the GEO database, and aberrantly methylated–differentially expressed (AMDE) genes were identified based on the relationship between methylation and expression. Furthermore, we conducted weighted correlation network analysis (WGCNA) and co-expression analysis to identify the core AMDE genes strongly involved in atherosclerosis. K-means clustering was used to characterize two subtypes of atherosclerosis in GSE40231, and then 29 samples were recognized as validation dataset (GSE28829). In a blood sample cohort (GSE90074), chi-square test and logistic analysis were performed to explore the clinical implication of the K-means clusters. Furthermore, significance analysis of microarrays and prediction analysis of microarrays (PAM) were applied to identify the signature AMDE genes. Moreover, the classification performance of signature AMDE gene-based classifier from PAM was validated in another blood sample cohort (GSE34822). A total of 1,569 AMDE mRNAs and eight AMDE long non-coding RNAs (lncRNAs) were identified by differential analysis. Through the WGCNA and co-expression analysis, 32 AMDE mRNAs and seven AMDE lncRNAs were identified as the core genes involved in atherosclerosis development. Functional analysis revealed that AMDE genes were strongly related to inflammation and axon guidance. In the clinical analysis, the atherosclerotic subtypes were associated with the severity of coronary artery disease and risk of adverse events. Eight genes, including *PARP15*, *SERGEF*, *PDGFD*, *MRPL45*, *UBR1*, *STAU1*, *WIZ*, and *LSM4*, were selected as the signature AMDE genes that most significantly differentiated between atherosclerotic subtypes. Ultimately, the area under the curve of signature AMDE gene-based classifier for atherosclerotic subtypes was 0.858 and 0.812 in GSE90074 and GSE34822, respectively. This study identified the AMDE genes (lncRNAs and mRNAs) that could be implemented in clinical clustering to recognize high-risk atherosclerotic patients.

## Introduction

Even though a marked reduction in atherosclerotic cardiovascular disease (CVD) mortality due to the application of new therapies has been observed, atherosclerosis and its consequent clinical manifestations are the leading causes of mortality worldwide (Nilsson, 2017). Recently, studies have revealed the underlying associations between epigenetics and atherosclerosis (Heo et al., 2016; Rizzacasa et al., 2019), suggesting a high molecular heterogeneity and pathogenic complexity in atherosclerosis, which still needs further sub-classification of atherosclerosis to improve diagnostic and treatment strategies.

Epigenetics is defined as the study of any potentially stable and, ideally, heritable change in gene expression or cellular phenotype that occurs without changes in Watson–Crick base-pairing of the DNA sequence (Goldberg et al., 2007). Epigenetic mechanisms include DNA methylation or demethylation, histone acetylation or deacetylation, and non-coding RNA regulation (Khyzha et al., 2017). In the past few years, increasing evidence has indicated that modulation of epigenetic mechanisms, especially methylation and long non-coding RNA (lncRNA) regulation, plays a pivotal role in the process of atherosclerosis (Tabaei and Tabaee, 2019).

Unsupervised clustering analysis is an agnostic multivariable method that is used to aggregate similar cases without the potentially confounding effects of pre-established diagnosis (Lancaster et al., 2019). This type of analysis has been widely applied to reveal epigenetic-related subgroups in cancer (Fiedler et al., 2019; Fukuoka et al., 2020). However, few studies revealed the different subgroups of atherosclerosis involved in the epigenetic process using machine learning approaches.

In the present study, we used integrative analysis to identify the aberrantly methylated–differentially expressed (AMDE) genes (mRNAs and lncRNAs) that could be used to define novel risk subgroups of atherosclerosis. Furthermore, using various statistical methods, we selected eight signature AMDE genes as the potential therapeutic targets for patients with high risk of atherosclerosis.

## Materials and Methods

### Study Datasets and Design

The four previously published gene expression profiles (GSE40231, GSE28829, GSE90074, and GSE34822) and one previously published gene methylation profile (GSE46394) were obtained from the Gene Expression Omnibus (GEO) database^1^.

**FIGURE 1:**
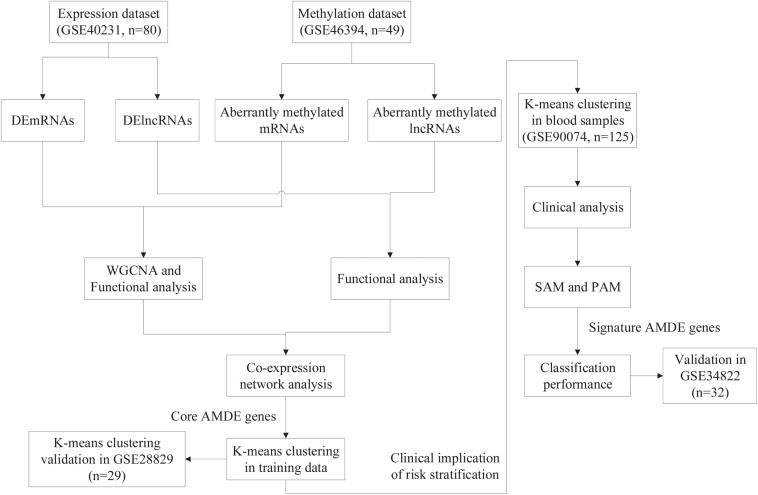
Flow chart of the entire study.

The overall design of the study is shown in Figure 1. For differential analysis, data collection from stable atherosclerotic and normal artery wall samples in GSE40231 [The Stockholm Atherosclerosis Gene Expression (STAGE) study] and GSE46394 [genome-wide DNA methylation aberrations in human atherosclerosis (450K)] were used to identify AMDE genes (including mRNAs and lncRNAs) (Hägg et al., 2009; Zaina et al., 2014). Furthermore, weighted correlation network analysis (WGCNA) and co-expression network analysis were applied to find the core AMDE genes. Moreover, K-means clustering analysis was utilized to identify different atherosclerotic clusters based on core AMDE genes in atherosclerotic samples in GSE40231 (training dataset). Then, we reapplied K-means clustering analysis in another atherosclerotic artery wall profile (GSE28829, gene expression in early and advanced atherosclerotic plaque from human carotid) as validation dataset (Döring et al., 2012). To explore the clinical implication of risk stratification, GSE90074 and GSE34822 datasets, including blood samples from atherosclerosis patients, were applied for further analytic procedures and considered as training and validation dataset, respectively. Clinical characteristics were compared between different clusters in GSE90074 (Ravi et al., 2017). Then, significance analysis of microarrays (SAM) and prediction analysis of microarrays (PAM) were used to choose signature AMDE genes as subtype classifier. Lastly, the classification performance of signature AMDE genes was validated in GSE34822 (transcriptome analysis in patients with progressive coronary artery disease) (Nührenberg et al., 2013).

### Data Preprocessing

The probes obtained from the GEO series were annotated into lncRNAs and mRNAs using GENCODE version 19 (equivalent to Ensembl GRCh37). After excluding the probes on sex chromosomes and with single nucleotide polymorphism (SNP), the valid CpG sites in the HM450K methylation microarrays were annotated as Ensembl transcript IDs which were then converted into gene IDs using the “*biomaRt*” package in R. Once several probes in the microarray were mapped to the same gene, the ultimate expression value of the gene was calculated as the mean value of several probe expressions. The combat function in the “sva” R package was applied to remove the batch effects, and the expression values were quantile-normalized across different biological samples.

### Differential Analysis

After excluding non-artery samples (*n* = 198), 40 atherosclerotic artery wall (AAW) samples and 40 non-atherosclerotic artery wall (NAAW) samples with expression profile from GSE40231 were used to identify the differentially expressed genes (DEGs), including differentially expressed mRNAs (DEmRNAs) and lncRNAs (DElncRNAs). The “limma” R package was used for DEG selection according to the following criteria: (a) adjusted *P*-value < 0.05 and (b) absolute log2 fold change > 1.

Thirty-four AAW and 15 NAAW samples with methylation profiles from GSE46394 were used to screen the aberrantly methylated genes (AMGs). Student *t*-test was used to check the difference in the beta values of the AAW and the NAAW samples according to the following criteria: (a) *P*-value < 0.05 and (b) | mean (AAW) – mean (NAAW)| > 0.1. Lastly, 11,995 hypermethylated CpG sites and 4,904 hypomethylated CpG sites were identified. Then, the aberrantly methylated CpG sites were mapped into corresponding genes for further accessing of AMDE genes.

According to the principle of the relationship between methylation and expression, hypomethylated–highly expressed genes were detected by overlapping the hypomethylated and upregulated genes, and hypermethylated–negligibly expressed genes were detected by overlapping the hypermethylated and downregulated genes (Wang et al., 2019; Zhao et al., 2020). Hypergeometric tests were used to calculate the *P* value of the overlapping results of AMGs with DElncRNAs and DEmRNAs.

### WGCNA

Different modules were constructed using the “WGCNA” package in R (Langfelder and Horvath, 2008). The modules significant for atherosclerosis development were identified by correlation analysis. The threshold for the determination of weighted adjacency matrix was fixed at a soft power of 12 and a scale-free *R*^2^ > 0.85, respectively. Then, the hubs were identified as the highest-degree nodes, and the adjacency matrix was converted as the topological overlap matrix (TOM). In TOM, the network connectivity of each gene was calculated and divided into modules with similar expression patterns through average linkage hierarchical clustering. Next, module–trait (NAAW and AAW) co-expression similarity and adjacency analyses were performed in six identified gene modules.

### Functional and Co-expression Analyses

Co-expression was predicted on the basis of expression of correlation coefficient (Pearson’s correlation > 0.9 or <-0.9) and statistical significance (*P* < 0.05), and then we extracted the protein-coding genes co-expressed with target lncRNAs for further functional annotation. We performed Gene Ontology (GO) enrichment and Kyoto Encyclopedia of Genes and Genomes (KEGG) pathway analyses of the lncRNAs and mRNAs in the most correlative modules using the Enrichr database (Kuleshov et al., 2016).

Furthermore, co-expression analysis of the AMDE lncRNAs and mRNAs was performed. Spearman’s analysis was performed to define significant gene pairs according to the following criteria: (a) | *r*| > 0.3 and (b) *P* value < 0.05. We then used the Cytoscape software to select the core genes (AMDE mRNAs and lncRNAs) in the co-expression network.

### K-Means Clustering of the Training (GSE40231) and Validation (GSE28829) Datasets

K-means clustering of unions of the core ADME genes was performed using the “ConsensusClusterPlus” package in R based on the AAW samples for risk stratification (Wilkerson and Hayes, 2010). In total, two to 10 K-means clusters were obtained based on the gene expression values. Consensus matrix heat maps and consensus cumulative distribution functions (CDF) were applied in ascertainment of the K optimizing clustering stability. Furthermore, to identify the relationship of K-means clusters from training (GSE40231) and validation datasets (GSE28829), “ModulePreservation” function from WGCNA package was used to validate the preservation of discovery clusters. Ultimately, K-means = 2 was selected as the optimal value for the training (GSE40231) and validation (GSE28829) datasets.

### Clinical Analysis

To clarify the clinical implication of the core AMDE genes in atherosclerotic progression, K-means clustering analysis based on the core AMDE genes was also performed with data from blood samples. Dataset GSE90074, including blood samples of atherosclerosis patients with complete clinical information, was chosen for further procedure, and data from GSE34822, also with blood samples, were selected as the validation cohort.

After excluding the patients with coronary artery disease (CAD) class equal to 0 (*n* = 18), 125 patients from phase 2 of the Supporting a Multi-disciplinary Approach to Researching Atherosclerosis study were included (GSE90074). The standards used to quantify CAD severity were consistent with corresponding publications. The clinical characteristics of each atherosclerosis subtype were identified and compared after K-means clustering. The logistic regression analysis of obstructive CAD with subtypes was also performed in both unadjusted and adjusted models.

### Significance Analysis of Microarrays

To identify the differential clusters of the core AMDE genes, we use SAM, which specifically compares high-throughput data such as microarray findings. SAM was used to determine whether the expression of each gene in the two given groups was significantly different. Hence, we used the *sam* function in “siggenes” package in R software to measure the expression of significant AMDE genes in the blood sample dataset (GSE90074) after K-means clustering (Tusher et al., 2001).

### Signature Selection and Validation Using a Classifier

We then inputted the SAM-prioritized significant AMDE genes to perform PAM, which uses the nearest shrunken centroid methodology, for the classification of K-means clusters in the blood sample dataset (GSE90074) (Tibshirani et al., 2002), and all the signature AMDE genes that best characterized each cluster were identified using the *pamr.cv* function of “pamr” package in R software.

Next, to validate the classifier performance of signature AMDE genes from PAM, GSE34822 was identified as the validation cohort. We calculated the sensitivity, specificity, precision, and accuracy in GSE90074 and repeat the whole process for 10 times (Mallik and Zhao, 2018), and then the classifier of signature AMDE genes from GSE90074 was applied in the validation dataset (GSE34822). Furthermore, the receiver operating characteristic (ROC) curves for K-means clusters in GSE90074 and GSE34822 were also plotted based on the PAM-derived classifier from GSE90074 using “pROC” package in R. All statistical analyses were performed using R software.

## Results

### Identifying the Aberrantly Methylated DEGs

**FIGURE 2:**
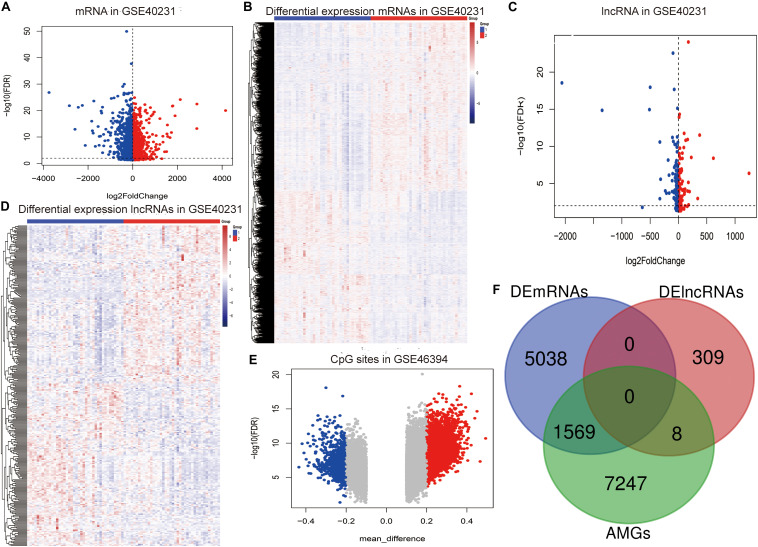
Identification of the aberrantly methylated–differentially expressed (AMDE) genes: **(A)** volcano plot of the differentially expressed mRNAs (DEmRNAs) in GSE40231, **(B)** heat map of DEmRNAs in GSE40231, **(C)** volcano plot of the differentially expressed lncRNAs (DElncRNAs) in GSE40231, **(D)** heat map of DElncRNAs in GSE40231, **(E)** volcano plot of the aberrantly methylated CpG sites in the GSE46394, and **(F)** Venn diagram showing the overlapping AMDE genes.

The flow chart of the entire study is shown in Figure 1, and the detailed information on these datasets is summarized in Supplementary Table 1. By comparing the AAW and the NAAW specimens from the GSE40231 profile, 6,607 DEmRNAs (3,137 upregulated and 3,470 downregulated) were identified (Figure 2A), and the expression levels of DEmRNAs among each sample are shown in Figure 2B. Moreover, 317 DElncRNAs (166 upregulated and 151 downregulated) were identified (Figure 2C), and the heat map of the DElncRNAs are shown in Figure 2D. Differentially methylated CpG sites were analyzed (Figure 2E). After being mapped onto the respective genes, 8,824 AMGs (5,879 hypermethylated and 2,945 hypomethylated genes) from the AAW and the NAAW samples were identified.

Subsequently, a total of 1,569 mRNAs and eight lncRNAs with a negative correlation between methylation and expression were recognized as the AMDE genes (Figure 2F), and the *P* values of hypergeometric tests in the overlapping analysis of DElncRNAs and DEmRNAs with AMGs were significant (both *P* values were <0.0001).

### WGCNA and Functional Analysis

**FIGURE 3:**
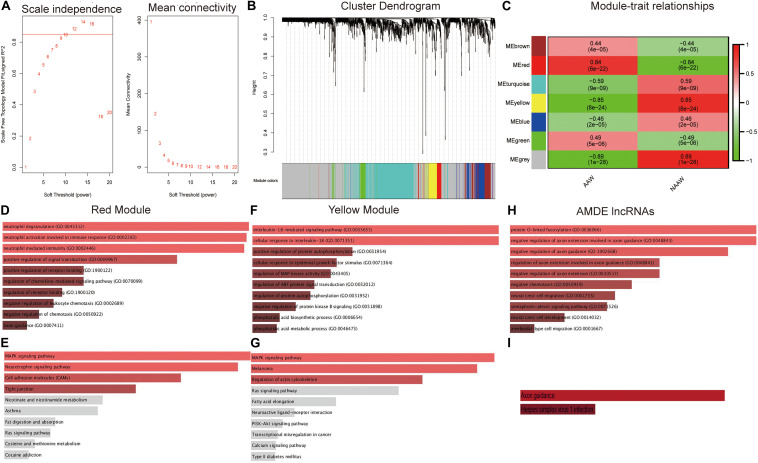
Weighted correlation network analysis (WGCNA) and functional analysis of the aberrantly methylated–differentially expressed (AMDE) genes: **(A)** scale-free networks of scale independence and mean connectivity, **(B)** clustering dendrogram of AMDE mRNAs obtained by WGCNA, **(C)** the relationship of each WGCNA module in the normal and atherosclerotic artery wall samples, **(D)** Gene Ontology (GO) analysis of the AMDE mRNAs in the red module, **(E)** Kyoto Encyclopedia of Genes and Genomes (KEGG) analysis of the AMDE mRNAs in the red module, **(F)** GO analysis of the AMDE mRNAs in the yellow module, **(G)** KEGG analysis of the AMDE lncRNAs in the yellow module, **(H)** GO analysis of the AMDE lncRNAs, and **(I)** KEGG analysis of the AMDE lncRNAs.

WGCNA was performed next to select AMDE mRNAs or lncRNAs in the most correlative modules with atherosclerosis. However, AMDE lncRNAs could not be conducted through WGCNA due to the low abundance of AMDE lncRNAs. According to the scale-free topology criteria, soft power 12 was chosen as the soft thresholding to set up a weighted adjacency matrix (Figure 3A). After calculating the module eigengenes, six modules were generated (Figure 3B). The red and yellow module eigengenes with lowest *P* values and highest correlative index were most related to the clinical atherosclerosis traits and analyzed in the further procedure (AAW and NAAW, respectively) (Figure 3C).

To obtain insight into the molecular function of these AMDE genes in atherosclerosis development, we performed GO enrichment and KEGG pathway analyses of the red and yellow AMDE mRNA modules and AMDE lncRNAs. The AMDE mRNAs in red modules showed a biological process of neutrophil degranulation, neutrophil activation involved in immune response, and neutrophil-mediated immunity (Figure 3D), and *MAPK* and neurotrophin signaling, respectively, were the most significant pathways for AMDE mRNAs in red modules (Figure 3E). Furthermore, the AMDE mRNAs in yellow modules were significantly involved in interleukin-18-mediated signaling pathway and cellular response to interleukin-18 process (Figure 3F), and Figure 3G also showed that *MAPK* signaling was the most significant pathway for AMDE mRNAs in yellow modules. The GO analysis also showed that the AMDE lncRNAs were involved in protein O-linked fucosylation, negative regulation of axon extension involved in axon guidance, and negative regulation of axon guidance (Figure 3H), whereas KEGG analysis revealed that their most significant molecular function was axon guidance (Figure 3I).

### Co-expression Network and K-Means Clustering in AAW Samples

**FIGURE 4:**
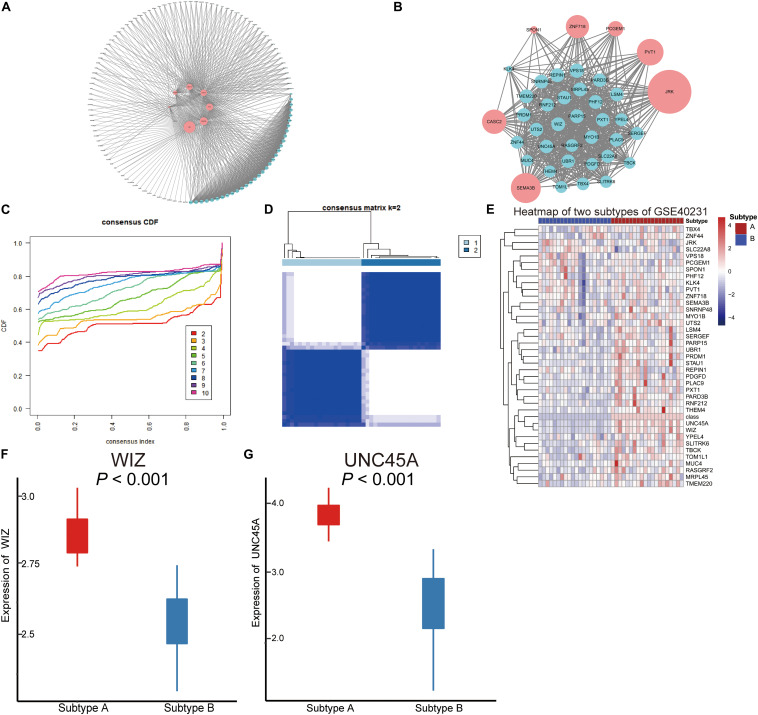
Co-expression network of the aberrantly methylated–differentially expressed (AMDE) genes and K-means clustering of GSE40231: **(A)** co-expression analysis of the AMDE lncRNAs and mRNAs in the red and yellow module, **(B)** co-expression network of the hub AMDE genes, **(C)** empirical cumulative distribution function plot of the *k* value ranging from 2 to 10 in GSE40231, **(D)** heat map of the consensus matrix for *k* = 2 in GSE40231, **(E)** heat map of the expression levels of the core AMDE genes in GSE40231, **(F)** box plot of *WIZ* expression in the different atherosclerosis subtypes (GSE40231), and **(G)** box plot of *UNC45A* expression in the different atherosclerosis subtypes (GSE40231).

Hub AMDE gene pairs were calculated by Spearman’s analysis (Figure 4A). To identify the core AMDE mRNAs and lncRNAs, node degree >10 was defined as the threshold. Ultimately, 32 mRNAs and seven lncRNAs were recognized as the core AMDE genes involved in the atherosclerotic process (Figure 4B).

Forty AAW samples from the training dataset (GSE40231) were chosen to further explore the core AMDE gene-associated subtypes by K-means consensus clustering, with the consensus distribution for each K value displayed using an empirical CDF plot (Figure 4C). Accordingly, we chose *k* = 2 as the optimal value due to the stable horizontal mid-portion between 0 and 1 and plotted the heat map of the consensus matrix for *k* = 2 (Figure 4D). Furthermore, the expression of the core AMDE genes for the different subtypes of atherosclerosis was plotted (Figure 4E). Lastly, *WIZ* and *UNC45A* were found to be the two DEGs mostly associated with two atherosclerosis subtypes (Figures 4F,G).

Furthermore, to verify that the two atherosclerosis subtypes were the optimal choices for K-means clustering, GSE28829 with 29 AAW samples was chosen as the validation profile. Consensus matrix heat maps for k-2, 3, and 4 (Supplementary Figures 1A–C), CDF plot (Supplementary Figure 1D), and tracking plot (Supplementary Figure 1E) indicated that K-means = 2 was recognized as the optimal value. Moreover, the preservation of discovery clusters was tested in K-means clusters of the validation dataset. The scatter plots of module membership showed positive correlations of the same subtype among different datasets (GSE40231 and GSE28829) (Supplementary Figures 2A,B). Furthermore, the network plots of the core AMDE genes in different subtypes were plotted (Supplementary Figures 2C–F).

### Clinical Implication of the Signature AMDE Genes in Blood Sample Datasets

**FIGURE 5:**
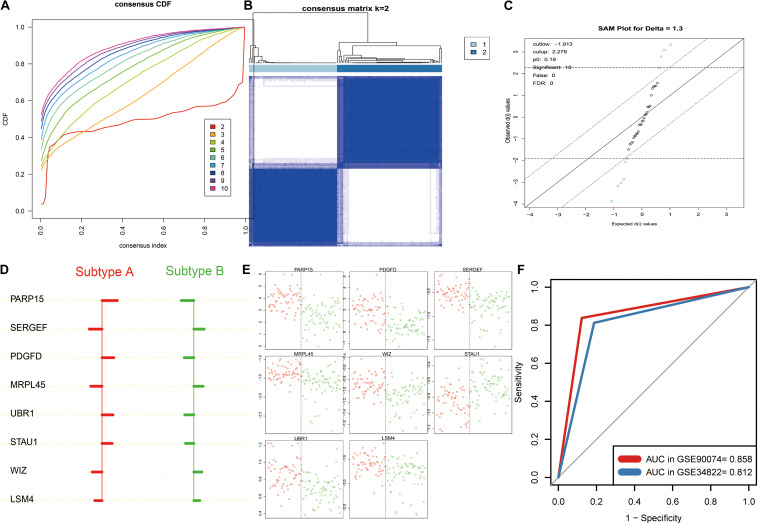
K-means clustering analysis and identification the signature aberrantly methylated–differentially expressed (AMDE) genes in the blood sample datasets: **(A)** empirical cumulative distribution function plot of the *k* value ranging from 2 to 10 in GSE90074, **(B)** heatmap of the consensus matrix for *k* = 2 in GSE90074, **(C)** using significance analysis of microarrays to identify the significant AMDE genes in the different atherosclerosis subtypes, **(D)** unique features (signature AMDE genes) of the different atherosclerosis subtypes obtained by prediction analysis of microarrays (GSE90074), **(E)** raw expression levels of the eight signature AMDE genes in the given specific threshold (GSE90074), and **(F)** receiver operating characteristic curves of the signature AMDE genes for atherosclerosis subtypes in GSE90074 and GSE34822.

To explore the clinical implication of K-means clustering based on 39 core AMDE genes, the profiles of 125 blood samples of atherosclerosis patients (93 with obstructive CAD and 33 with no obstructive CAD) from the GSE90074 profile were included in the validation dataset. The CDF plot and the heat map of the consensus matrix showed that the two clusters of atherosclerotic blood samples still had better stable consensus distribution (Figures 5A,B).

Table 1 illustrates the baseline characteristics of the two atherosclerosis subtypes, indicating a higher prevalence of obstructive CAD (*P* = 0.028) in subtype B patients. In addition, subtype B patients, compared to subtype A patients, showed significantly more severe coronary atherosclerotic burden (*P* = 0.005). They were also thinner than subtype A patients (*P* = 0.031), suggesting a higher risk of CVD events. Moreover, logistic regression analysis showed a strong correlation between obstructive CAD and atherosclerotic subtypes even after adjustment (*P* = 0.022) (Table 2). Additionally, we used the partitioning around medoids algorithm to re-cluster the same dataset (GSE90074) and observed that these clusters, compared to the K-means clusters, showed a relatively poor discriminatory ability compared with K-means clusters (Supplementary Table 2).

**TABLE 1:** Baseline characteristics of validation data by subtype of atherosclerosis.

Characteristics		Subtype A (*n* = 57)	Subtype B (*n* = 68)	*P* value
Male sex		33 (58%)	37 (54%)	0.72
White race		40 (70%)	57 (84%)	0.08
Diabetes		27 (47%)	22 (32%)	0.10
Hyperlipidemia		43 (75%)	53 (78%)	0.83
Hypertension		48 (84%)	62 (91%)	0.28
Body mass index, kg/m^2^		29.5 (25.8–33.7)	27.0 (24.6–30.5)	0.031
Obstructive CAD		37 (65%)	56 (82%)	0.028
CAD class	1	20 (35%)	12 (18%)	0.005
	2	15 (26%)	16 (24%)	
	3	14 (25%)	12 (18%)	
	4	8 (14%)	28 (41%)	

*CAD, coronary artery disease.*

**TABLE 2:** Binary logistic regression analysis of subtype and obstructive coronary artery diseases.

Variable	Odd ratio	95% CI	*P* value
Univariate analysis			
Subtype B/A	2.52	1.10–5.77	0.028
Model 1^*a*^			
Subtype B/A	2.86	1.16–7.03	0.022
Gender (male)	2.88	1.12–7.42	0.028

*^*a*^Adjusted for gender, hypertension, hyperlipidemia, race, diabetes, and body mass index.*

Furthermore, SAM was performed, revealing that 10 AMDE genes (out of the 39 core AMDE genes) were most significantly differentiated K-means-derived atherosclerotic subtypes (Figure 5C). Moreover, we performed PAM and determined the unique features (AMDE genes) of the different atherosclerosis subtypes (Figure 5D). *PARP15*, *SERGEF*, *PDGFD*, *MRPL45*, *UBR1*, *STAU1*, *WIZ*, and *LSM4* were ultimately selected as signature AMDE genes, and the raw expression levels of these eight unique resultant genes in the given specific threshold were plotted (Figure 5E).

**FIGURE 6:**
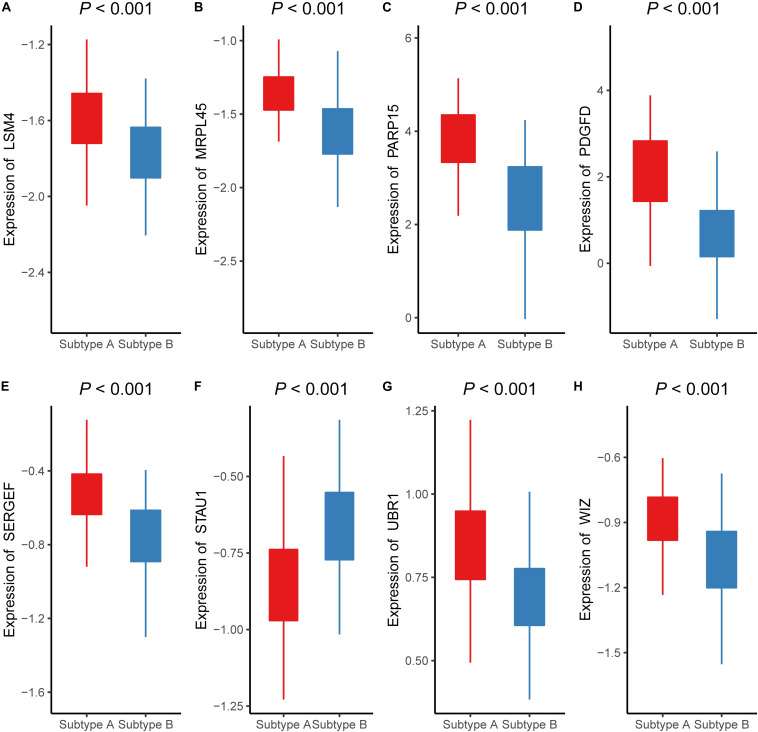
The expressions of eight signature aberrantly methylated-differentially expressed genes in different atherosclerosis subtype (GSE90074). The expression levels of **(A)** LSM4, **(B)** MRPL45, **(C)** PARP15, **(D)** PDGFD, **(E)** SERGEF, **(F)** STAU1, **(G)** UBR1, **(H)** WIZ in different subtypes of atherosclerosis.

To validate the classifier performance for the atherosclerosis subtypes based on eight signature AMDE genes from PAM analysis, GSE34822 was involved in the validation procedure. We plotted the shrunken subtype centroids for atherosclerosis subtype and the raw expression of eight signature AMDE genes of different atherosclerosis subtypes in GSE34822 (Supplementary Figure 3). Moreover, the sensitivity, specificity, precision, and accuracy for the K-means subtypes were calculated by 10-fold cross-validation of the signature AMDE gene-based classifier and repeated 10 times in GSE90074 (Table 3), and then this classifier was used in GSE34822 (Supplementary Table 3). Accordingly, the areas under the ROC curve (AUCs) of the classifier from GSE90074 for atherosclerosis subtypes were obtained in the same testing and testing process, suggesting the good distinguishable ability of the classifier (Figure 5F). Furthermore, the expression of these eight signature AMDE genes in the different atherosclerosis subtypes is shown in Figure 6.

**TABLE 3:** Classification performance of the resultant gene signature having all the features and samples for GSE90074.

Evaluation criteria	Average (SD)
Sensitivity	0.868 (0.27%)
Specificity	0.826 (0.22%)
Precision	0.855 (0.20%)
Accuracy	0.848 (0.30%)

## Discussion

The role of methylation in the pathophysiology of atherosclerosis has drawn increasing attention, and it is currently believed that investigating the improved insight on the methylation landscape on atherosclerosis development is pivotal for understanding the disease (Grimaldi et al., 2015). Recently, studies in patients with atherosclerosis have revealed a unique methylation profile (Zaina and Lund, 2014) and suggested that many factors, including shear stress, inflammation, oxidative stress, hyperhomocysteinemia, aging, and low-density lipoprotein oxidation, can initiate atherosclerosis-related methylation (Chan et al., 2004; Yideng et al., 2007; Steucke et al., 2015; Lee and Chiu, 2019). In particular, it was demonstrated that the protein-coding genes *KLF-2*, *KLF-4*, *HoxA5*, *ABCA1*, and *DDAH2* are hypermethylated and down-regulated, whereas *PDGF*, *MCP-1*, *LDLR*, *LOX-1*, and *BAX* are hypomethylated and up-regulated in a DNA methyltransferase (DNMT)-dependent manner during atherosclerosis progression (Dunn et al., 2014, 2015; Rizzacasa et al., 2019). The lncRNA-coding gene, cyclin-dependent kinase inhibitor 2B antisense RNA 1 (*CDKN2B-AS1*), can bind to *DNMT1*, thereby enhancing the methylation of the A disintegrin and metalloprotease 10 (*ADAM10*) promoter, leading to the suppression of the atherosclerotic inflammatory response and the promotion of cholesterol efflux in THP-1 macrophages (Li et al., 2019). In addition, the knockdown of lncRNA growth arrest-specific 5 (*GAS5*) promoted ATP-binding cassette transporter A1 (*ABCA1*) expression by inhibiting the methylation of the *ABCA1* promoter region, hence enhancing cholesterol reverse-transportation and reducing intracellular lipid accumulation (Meng et al., 2020). Despite these evidences, only few studies have illustrated the regulatory relationship between methylation and lncRNA-coding genes in atherosclerosis. Therefore, we conducted an integrative analysis of methylation and genes (including lncRNA and protein-coding genes) to explore the genome methylation conditions.

Most differential CpG sites were found to be hypermethylated in atherosclerosis samples (11,995 hypermethylated *vs*. 4,904 hypomethylated), which is in agreement with the findings of previous studies (Rangel-Salazar et al., 2011; Zaina et al., 2014). A subsequent functional analysis further revealed that AMDE genes in the red and yellow modules played pivotal roles in the inflammatory processes, including neutrophil activation, neutrophil-mediated immunity, and cellular response to IL-18. Additionally, the KEGG pathway analysis indicated that *MAPK* signaling could be the key pathway underlying methylation abnormalities in atherosclerosis. Furthermore, AMDE lncRNAs were strongly associated with axon guidance and the semaphorin–plexin signaling pathway. Interestingly, semaphorins were originally identified for their role in the axon guidance pathway during the embryonic development of the nervous system (Van Agtmael et al., 2010). Interestingly, emerging evidence has also suggested that the genes of the axon guidance pathway are related to atherosclerosis. For instance, *Sema3A*, one of the members of the semaphorin family, has been shown to induce the apoptosis of endothelial cells and monocyte-derived macrophages (Guttmann-Raviv et al., 2007; Moretti et al., 2008; Vadasz et al., 2013).

To validate the K-means clusters from the training dataset, we conducted K-means clustering analysis in the validation dataset (GSE28829). Both GSE40231 and GSE28829 included tissue samples from a large (aortic and carotid) artery plaque and measured the global gene expression based on the same platform (GPL570). The “ModulePreservation” function of WGCNA package revealed that the K-means clusters from different datasets had similar core AMDE genes expressions. To also investigate the clinical implication of K-means clustering, we also conducted K-means clustering in the blood sample dataset (GSE90074), which was a more easily obtained tissue in atherosclerosis patients. CAD is a severe form of atherosclerosis; however, current techniques for directly identifying the high risk of CAD patients are restricted to coronary computer tomography, angiography, and intravascular ultrasound. Our results showed that the K-means clusters were associated with coronary atherosclerotic burden in GSE90074, including the data of atherosclerosis patients with CAD class > 0 (*n* = 125). To further construct a signature AMDE gene-based classifier for atherosclerotic subtypes, SAM and PAM were implemented to select the signature AMDE genes. Eight signature AMDE gene-derived classifiers showed an outstanding discriminative ability for atherosclerosis subtypes in both GSE90074 and GSE34822. Hence, detection the eight signature AMDE genes expression in a clinical situation could contribute to risk stratification of atherosclerosis patients.

Obesity has been considered a risk factor for CVD for several decades (Van Gaal et al., 2006). However, subtype B patients, who showed much worse coronary atherosclerotic severity, were found to be less likely to develop obesity. Hence, we assumed that subtype B patients were more vulnerable to atherosclerosis-related CVD events, such as obstructive CAD. Therefore, these high-risk patients could benefit from early appropriate medication, including statins and anti-platelet drugs.

The variation in the different atherosclerosis subtypes could be explained by the signature AMDE genes significantly involved in atherosclerosis progression. Among these genes, only *STAU1*, *SERGEF*, and *PDGFD* are known to be associated with atherosclerosis. Microvesicles containing *STAU1*-microRNA were found to significantly delay atherosclerosis development by mitigating dyslipidemia, hypertension, and heart wall remodeling (Alexandru et al., 2020). In addition, large cohort genome-wide admixture and association studies demonstrated that SNPs in *SERGEF* gene region were strongly associated with common carotid artery intima-media thickness (Shendre et al., 2017). Several studies have demonstrated that *PDGFD* plays a vital role in atherosclerosis development (Karvinen et al., 2009; Lee and Li, 2018). Moreover, *PDGFD* mRNA and protein expression were induced after the differentiation of THP-1 monocytes into macrophages, while *PDGFD* enhanced matrix metalloproteinase-2 mRNA expression in a concentration-dependent manner (Wagsater et al., 2009). Furthermore, the secretion of *PDGFD* by macrophages could attenuate the expression of smooth muscle (SM) α-actin, SM-myosin heavy chains in smooth muscle cells, and phenotypic conversion (Thomas et al., 2009). Additionally, a genome-wide association study revealed that SNPs in the *PDGFD* locus are related to CAD (Coronary Artery Disease (C4D) Genetics Consortium, 2011).

Although many interesting points have been demonstrated in our study, there are still some limitations. Firstly, more homogenous samples for methylation and transcription datasets should be obtained in a further study to more precisely identify the genes involved in the atherosclerotic development. Secondly, the atherosclerosis development-related CpG sites, especially in the promoter and the enhancer regions of the genes, should be further demonstrated.

In conclusion, the expression and the methylation microarray analyses of mRNAs and lncRNAs were performed, and then K-means clustering analysis revealed high-risk atherosclerosis subtypes, which are characterized by AMDE genes. Eight signature AMDE genes were recognized as potential key molecules in atherogenesis progression and could be used as the classifier for risk stratification for atherosclerosis patients. Nevertheless, further *in vitro* and *in vivo* studies are required to provide additional details on the molecular mechanisms of the underlying role of the eight signature AMDE genes in atherosclerosis development and progression.

## Web of Resources

Enrichr database: https://maayanlab.cloud/Enrichr/

Cytoscape software: https://cytoscape.org/

R software: https://www.r-project.org/

Venn diagram online programs: http://bioinformatics.psb.ugent.be/webtools/Venn/

## Data Availability Statement

Publicly available datasets were analyzed in this study. This data can be found here: https://www.ncbi.nlm.nih.gov/geo/. And the detailed information has been listed in Supplementary Tables 1, 2.

## Author Contributions

YX and SL drafted the manuscript. JS revised the manuscript critically. WZ, JX, YZ, and ZX downloaded and analyzed the data. JS, YX, and YG designed the study. JS revised the final version of the manuscript. All the authors read and approved the final version of the manuscript.

## Conflict of Interest

The authors declare that the research was conducted in the absence of any commercial or financial relationships that could be construed as a potential conflict of interest.

## Footnotes

1 https://www.ncbi.nlm.nih.gov/geo

## Supplementary Material

Supplementary Figure 1 Measuring consensus and determining the number of subtypes (optimal *k*) in the validation dataset (Gse28829). Heat map of the consensus matrix for **(A)** *k* = 2, **(B)** *k* = 3, and **(C)** *k* = 4. **(D)** Empirical cumulative distribution functio plot of the *k* value ranging from 2 to 10 in Gse28829. **(E)** Tracking plot of the *k* value ranging from 2 to 10 in Gse28829.

Supplementary Figure 2 Validation of the preservation of the discovery clusters in the validation dataset. The two clusters in Gse40231 were identified as “Gse40231 subtypeA” and “Gse40231 subtypeB.” The two clusters in Gse28829 were also identified as “Gse28829 subtypeA” and “Gse28829 subtypeB.” Then, we calculated the module eigengene of core Amde genes in each subtype and then generated the module membership (Kme). **(A)** Scatter plots of Kme in Gse40231 subtypeA vs. Gse28829 subtypeA, **(B)** scatter plots of Kme in Gse40231 subtypeB vs. Gse28829 subtypeB; network plot of the module of core aberrantly methylated–differentially expressed genes in **(C)** Gse28829 subtypeA, **(D)** Gse40231 subtypeA, **(E)** Gse40231 subtypeB, and **(F)** Gse28829 subtypeB.

Supplementary Figure 3 Prediction analysis of microarrays of signature aberrantly methylated–differentially expressed (Amde) genes in blood samples (Gse34822). **(A)** Signature Amde genes of the different atherosclerosis subtypes obtained by prediction analysis of microarrays (Gse34822) and **(B)** raw expression levels of the eight signature Amde genes in the given specific threshold (Gse34822).

Supplementary Figure 4 The first 100 differentially expressed genes between Aaw and Naaw samples (Gse40231).

Supplementary Table 1 Group information of Gene Expression Omnibus datasets.

Supplementary Table 2 Baseline characteristics of validation data by subtype (pam clusters).

Supplementary Table 3 Classification performance of the resultant gene signature having all the features and samples for Gse34822.

Supplementary Table 4 Sample information of each Gene Expression Omnibus accession (Gse40231, Gse46394, Gse28829, Gse90074, and Gse34822) used in the study.

Supplementary Table 5 Aberrantly methylated–differentially expressed (Amde) genes (including eight Amde lncRnas and 1,569 Amde mRnas).

Supplementary Table 6 Core aberrantly methylated–differentially expressed genes and their information of the node degree through a co-expression network analysis.

Supplementary Table 7 Subtype information of Gse40231 and Gse90074.

Supplementary Table 8 Expressions of core Amde genes in atherosclerostic samples of Gse40231.

Supplementary Table 9 Information on the aberrantly methylated CpG sites of the eight signature aberrantly methylated–differentially expressed genes.
