# Supplementary figures and images for: Aberrantly Methylated-Differentially Expressed Genes Identify Novel Atherosclerosis Risk Subtypes
Source: Front Genet. 2020 Dec 14;11:569572. doi: 10.3389/fgene.2020.569572 (PMC7767999; doi:10.3389/fgene.2020.569572)

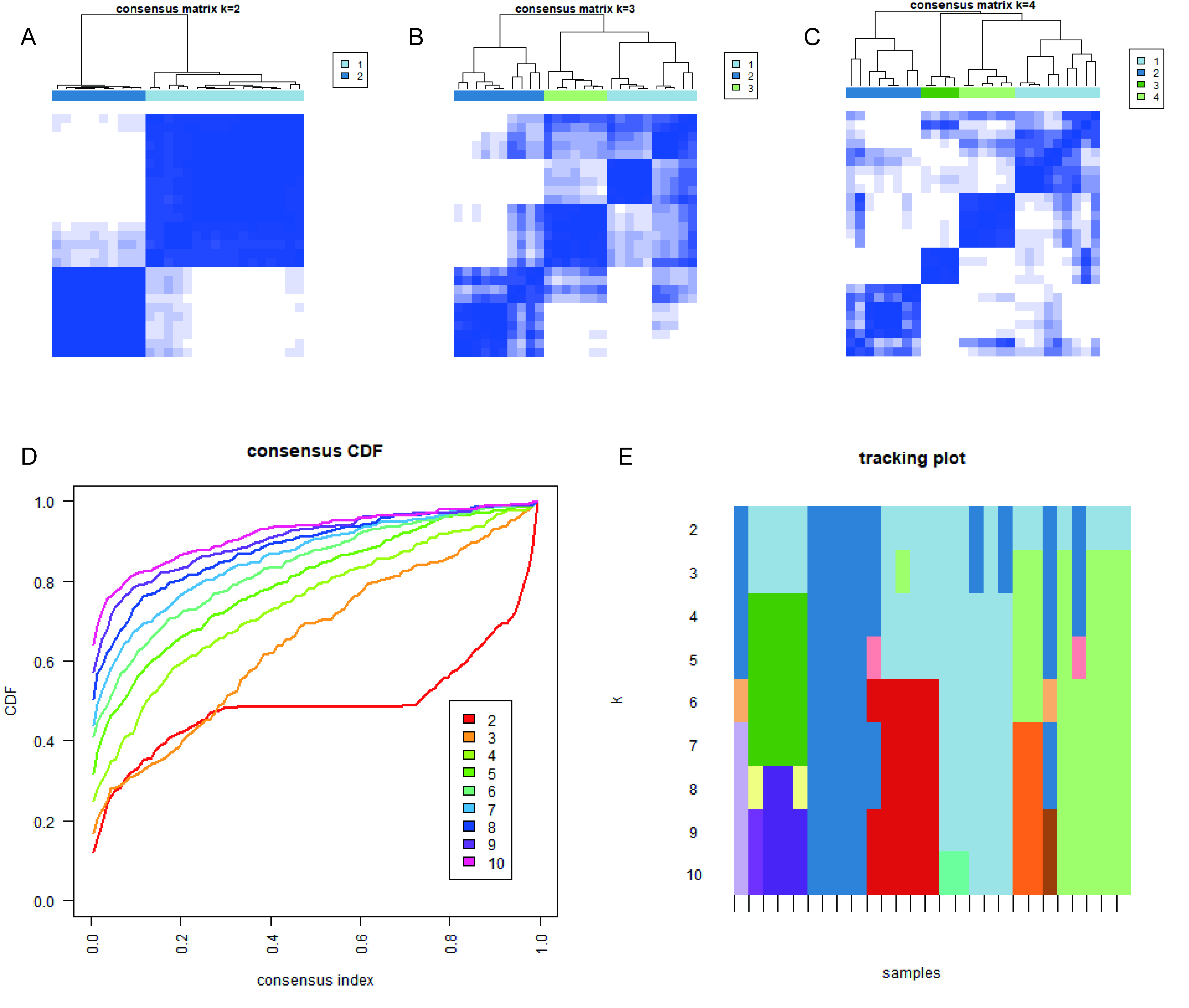

Supplement: Supplementary Figure 1 — Measuring consensus and determining the number of subtypes (optimal k) in the validation dataset (Gse28829). Heat map of the consensus matrix for (A) k = 2, (B) k = 3, and (C) k = 4. (D) Empirical cumulative distribution functio plot of the k value ranging from 2 to 10 in Gse28829. (E) Tracking plot of the k value ranging from 2 to 10 in Gse28829. [file Image_1.TIF]

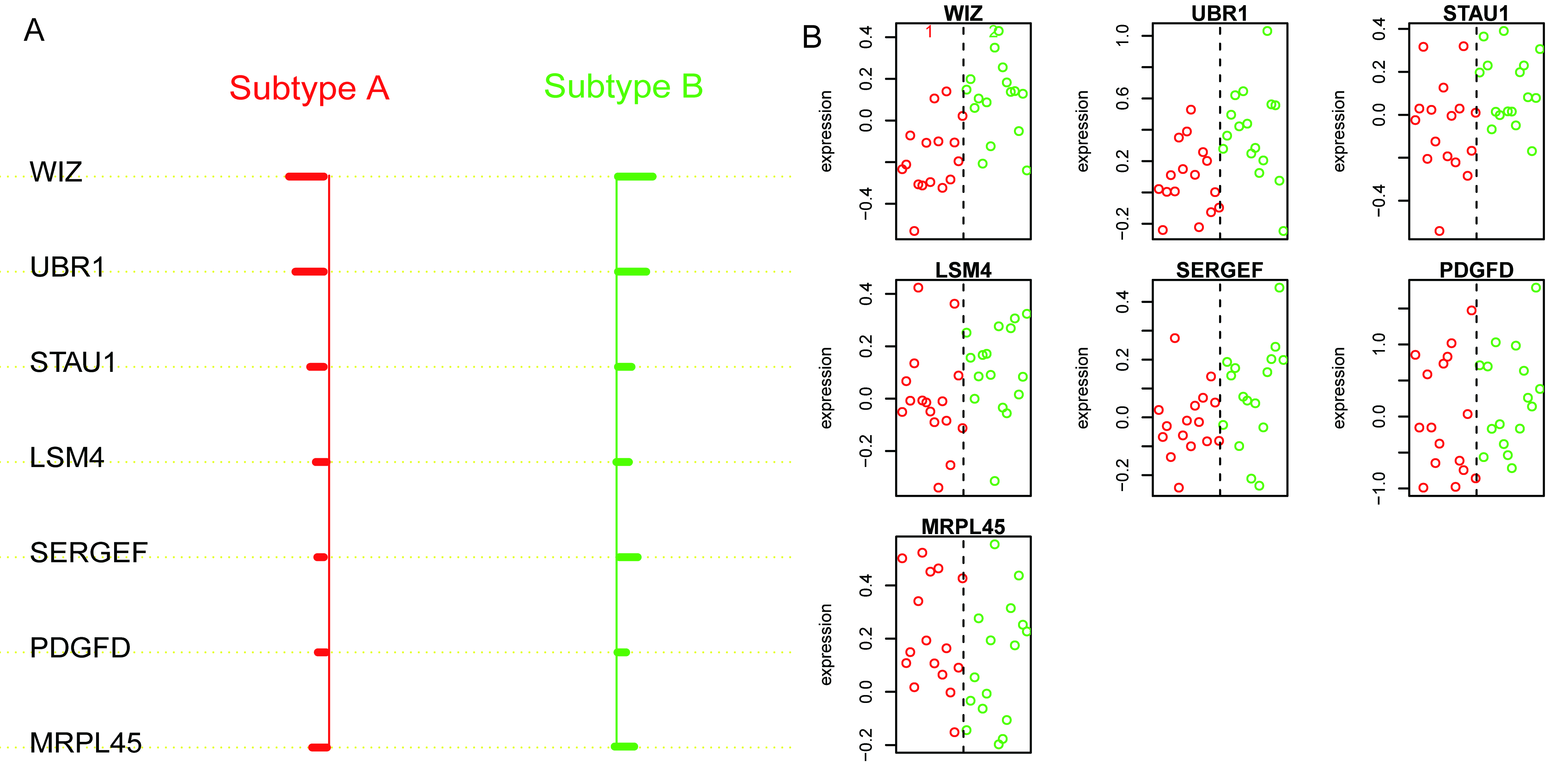

Supplement: Supplementary Figure 3 — Prediction analysis of microarrays of signature aberrantly methylated–differentially expressed (Amde) genes in blood samples (Gse34822). (A) Signature Amde genes of the different atherosclerosis subtypes obtained by prediction analysis of microarrays (Gse34822) and (B) raw expression levels of the eight signature Amde genes in the given specific threshold (Gse34822). [file Image_3.TIF]

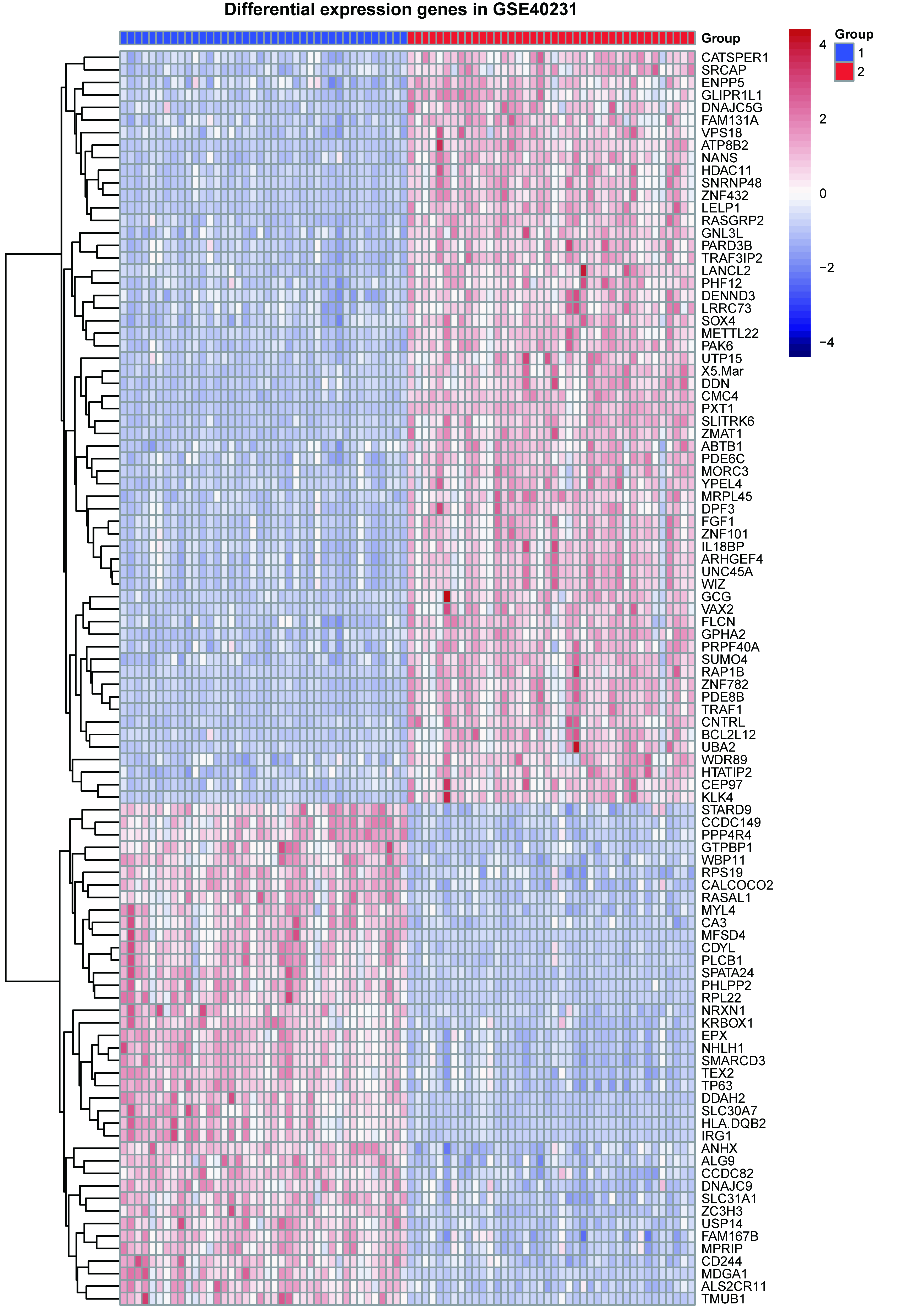

Supplement: Supplementary Figure 4 — The first 100 differentially expressed genes between Aaw and Naaw samples (Gse40231). [file Image_4.TIF]
