# Supplementary material for: Aberrantly Methylated-Differentially Expressed Genes Identify Novel Atherosclerosis Risk Subtypes
Source: Front Genet. 2020 Dec 14;11:569572. doi: 10.3389/fgene.2020.569572 (PMC7767999; doi:10.3389/fgene.2020.569572)
Supplement: Supplementary Table 2 — Baseline characteristics of validation data by subtype (pam clusters). [file Data_Sheet_2.DOCX]

Supplementary table 2: Baseline characteristics of validation data by subtypes (pam clusters)

| Characteristics | | Subtype A (n=77) | Grsubtype B (n=48) | P value |
| --- | --- | --- | --- | --- |
| Male sex | | 47 (61%) | 23 (48%) | 0.19 |
| White race | | 55 (71%) | 42 (88%) | 0.047 |
| Diabetes | | 31 (40%) | 18 (38%) | 0.85 |
| Hyperlipidemia | | 58 (75%) | 38 (79%) | 0.67 |
| Hypertension | | 65 (84%) | 45 (94%) | 0.16 |
| Body mass index, kg/m^2^ | | 28.25 (25.16-31.95) | 28.02 (24.63-32.36) | 0.62 |
| Obstructive CAD | 1 | 53 (69%) | 40 (83%) | 0.092 |
| CAD class | 1 | 24 (31%) | 8 (17%) | 0.057 |
|  | 2 | 17 (22%) | 14 (29%) |  |
|  | 3 | 19 (25%) | 7 (15%) |  |
|  | 4 | 17 (22%) | 19 (40%) |  |

Pam: partitioning around medoid
