# Supplementary material for: Aberrantly Methylated-Differentially Expressed Genes Identify Novel Atherosclerosis Risk Subtypes
Source: Front Genet. 2020 Dec 14;11:569572. doi: 10.3389/fgene.2020.569572 (PMC7767999; doi:10.3389/fgene.2020.569572)
Supplement: Supplementary Table 3 — Classification performance of the resultant gene signature having all the features and samples for Gse34822. [file Data_Sheet_3.DOCX]

Supplementary Table 3. Classification performance of the resultant gene signature having all the features and samples for GSE34822

| Evaluation criteria | Average (SD) |
| --- | --- |
| Sensitivity | 0.813 |
| Specificity | 0.813 |
| Precision | 0.813 |
| Accuracy | 0.815 |
